# Cross-Cultural Biases of Emotion Perception in Music

**DOI:** 10.3390/brainsci15050477

**Published:** 2025-04-29

**Authors:** Marjorie G. Li, Kirk N. Olsen, William Forde Thompson

**Affiliations:** 1 School of Psychological Sciences, Macquarie University, Macquarie Park, NSW 2109, Australia; marjorie.li@hdr.mq.edu.au; 2 Australian Institute of Health Innovation, Macquarie University, Macquarie Park, NSW 2109, Australia; kirk.olsen@mq.edu.au; 3 Faculty of Society and Design, Bond University, Gold Coast, QLD 4229, Australia

**Keywords:** emotion perception, emotional intensity, agitation, perceptual bias, cultural familiarity, musical features

## Abstract

**Objectives:** Emotion perception in music is shaped by cultural background, yet the extent of cultural biases remains unclear. This study investigated how Western listeners perceive emotion in music across cultures, focusing on the accuracy and intensity of emotion recognition and the musical features that predict emotion perception. **Methods**: White-European (Western) listeners from the UK, USA, New Zealand, and Australia (*N* = 100) listened to 48 ten-second excerpts of Western classical and Chinese traditional bowed-string music that were validated by experts to convey happiness, sadness, agitation, and calmness. After each excerpt, participants rated the familiarity, enjoyment, and perceived intensity of the four emotions. Musical features were computationally extracted for regression analyses. **Results**: Western listeners experienced Western classical music as more familiar and enjoyable than Chinese music. Happiness and sadness were recognised more accurately in Western classical music, whereas agitation was more accurately identified in Chinese music. The perceived intensity of happiness and sadness was greater for Western classical music; conversely, the perceived intensity of agitation was greater for Chinese music. Furthermore, emotion perception was influenced by both culture-shared (e.g., timbre) and culture-specific (e.g., dynamics) musical features. **Conclusions**: Our findings reveal clear cultural biases in the way individuals perceive and classify music, highlighting how these biases are shaped by the interaction between cultural familiarity and the emotional and structural qualities of the music. We discuss the possibility that purposeful engagement with music from diverse cultural traditions—especially in educational and therapeutic settings—may cultivate intercultural empathy and an appreciation of the values and aesthetics of other cultures.

## 1. Introduction

Music is experienced across all societies and conveys emotions ranging from basic emotions like happiness and sadness to more complex emotions like awe and triumph [1]. However, cultural background influences how people perceive these emotions, introducing biases in emotion perception [2]. These biases affect emotional judgements in perceiving emotion and experiencing emotion [3,4]. This study focused on perceived emotion, investigating how Western listeners perceive emotions in both familiar and unfamiliar musical traditions. Understanding how people emotionally respond to music from diverse cultures is essential for promoting cultural appreciation and understanding [5,6], as emotion perception underlies empathy, emotional intelligence, and social connection through a shared emotional space that transcends cultural boundaries [7,8,9,10,11]. This shared space allows listeners to engage with emotions in response to unfamiliar cultural traditions.

Previous research has demonstrated that familiarity with the music of one’s own culture enhances both the recognition of intended emotions and the perceived intensity of those emotions, as listeners are highly attuned to emotional cues within musical systems that they know well [12,13]. However, it remains unclear whether this advantage applies uniformly across different types of emotions. This investigation addresses that gap through three aims: (1) to examine whether Western listeners demonstrate greater accuracy in recognising emotions in familiar Western music compared to unfamiliar Chinese music; (2) to investigate whether Western listeners rate emotional intensity greater for emotions conveyed in Western music than in Chinese music; and (3) to assess whether musical features influence emotion recognition accuracy and perceived emotional intensity across these two musical traditions.

### 1.1. Emotion Recognition Across Musical Cultures

How do people recognise emotions expressed by music? Emotion perception in cross-cultural music is influenced by both psychophysical and culture-specific cues [14]. According to Juslin’s lens model [15], emotion recognition in music occurs through an additive and flexible process engaging multiple musical cues to influence emotional judgements. Emotion perception involves three stages. First, cue abstraction identifies and extracts auditory cues from the music [16]. Universal cues (e.g., tempo and loudness) are recognised similarly across cultures, while culture-specific cues (e.g., pentatonic scales in East Asian music) are unique to a particular tradition. Second, comparison and matching involve recognising patterns in the extracted cues and matching them with internalised emotional prototypes and stereotypes of emotion in music [17,18]. Third, judgement of emotion involves emotional appraisal based on the interaction of available cues [19]. In familiar music, both psychophysical and culture-specific cues contribute to more nuanced, intense, and accurate emotional perceptions, leading to judgement advantages. In unfamiliar music, although redundant universal cues support recognition when cultural knowledge is lacking, this unfamiliarity can evoke uncertainty and potentially trigger biases.

### 1.2. Dimensional Approaches to Emotion

The circumplex model of affect [20] organises emotions along two dimensions: valence (pleasantness) and arousal (intensity). Emotions like happiness (high arousal/positive valence), sadness (low arousal/negative valence), agitation (high arousal/negative valence), and calmness (low arousal/positive valence) occupy four quadrants. This model suits cross-cultural research, as music often conveys affect through these dimensions rather than discrete labels [21]. While happiness and sadness are commonly studied [13,22,23,24], agitation remains unexplored. Associated with musical tension, agitation involves feelings of nervousness, impatience, and irritation. Unlike fear or anger, which are typically directed toward an external threat or source of aggression, agitation is often an internally focused emotion, reflecting an unsettled or restless internal state. Research shows that agitation in music elicits physiological responses like increased heart rate, aligning it with other high-arousal emotions while differentiating it by its negative valence [25]. Its position opposite calmness on both arousal and valence axes makes it a compelling target for study. Previous research found Western listeners could recognise agitation in both Western classical and Chinese music with above-chance accuracy, but they exhibited response bias that misjudged Chinese music as Western music [26]. This cultural bias in classification judgement makes agitation an important yet understudied emotion in cross-cultural music research.

### 1.3. Musical Features in Emotion Recognition

Musical features, such as tempo, loudness, and complexity, shape emotion perception. For example, early research investigating Western listeners’ interpretation of emotional meaning in Indian Hindustani ragas laid the foundation for the Cue-Redundancy Model [22]. This model posits that emotion recognition in unfamiliar music from other cultures is significantly underpinned by psychophysical cues such as tempo (pace), pitch height, timbre, and intensity. Musical features such as loudness, tempo, and complexity likely contribute to the perception of high-arousal emotions, as they align with natural biomechanical and physiological arousal responses [22]. These features predict the experience of anger [27] and may play a similar role in perceived agitation. Cultural differences shape how acoustic cues convey emotion, highlighting both universal and culture-specific musical signals [13,28,29]. However, their roles in expressing high-arousal negative emotions (e.g., agitation), particularly within unfamiliar musical traditions, remains unexamined. This study explored how these features influence perceived agitation in familiar Western and unfamiliar Chinese music and how agitation differs from other emotions within the circumplex model, such as happiness, calmness, and sadness. Specifically, we asked whether cross-cultural perceptual biases shape the perception of these emotions in unfamiliar music.

### 1.4. Perceptual Bias in Emotion Perception Across Musical Cultures

Perceptual bias refers to Western listeners recognising specific emotions more accurately and more intensely in music from familiar traditions. This bias likely arises from cultural differences in how musical structures are appraised [5,19,30,31]. One form of perceptual bias is the in-culture advantage—a tendency for listeners to judge emotions in music from their own cultural tradition more accurately than music from unfamiliar ones, likely due to enculturation and prior exposure to that cultural system [2,12,13]. This advantage is evident from neuroimaging research, which indicates that familiar music, compared to unfamiliar music, more robustly activates emotion-related and reward-processing brain regions [32] and elicits distinct theta synchronisation patterns associated with pleasantness perception [33]. These findings suggest that cultural familiarity enhances emotional engagement and facilitates efficient emotional processes.

While an in-culture advantage in recognition has been observed for some emotions, it remains unclear whether negative emotions such as agitation follow the same pattern. Unfamiliar music tends to trigger uncertainty and unpredictability, leading to repeated violations of musical expectations [34,35]. Violations in expectations, in turn, might amplify the perception of agitation, increasing the chance that it will be detected in music from another culture.

### 1.5. The Present Study

This study aimed to investigate how Western listeners perceive emotions in response to Western classical and Chinese traditional music by focusing on recognition accuracy and perceived intensity, as well as the predictive value of musical features. Happiness, sadness, agitation, and calmness were selected based on their emotional qualities within the arousal–valence model [20,36]. Participants classified each excerpt as Western or Chinese in a forced-choice task and rated their familiarity, enjoyment, and perceived emotions. Musical features were computationally extracted for regression analyses. The study adopted a 2 (culture: Western, Chinese) × 4 (intended emotion: agitation, happiness, sadness, calmness) within-subjects design. While cultural familiarity typically enhances emotion recognition [12,13], these advantages should depend on the emotion. Basic emotions such as happy, sad and calm should be well recognised in culturally familiar music. However, agitation may be more salient for unfamiliar music, given that agitation often arises when expectations are violated [34,35] and familiar cues are absent [14].

Based on these considerations, three predictions were made. First, Western listeners should recognise emotions more accurately in familiar Western classical music than in unfamiliar Chinese traditional music, particularly for happiness, sadness, and calmness, but not for agitation. Second, Western listeners should perceive greater emotional intensity in Western classical music than in Chinese music, particularly for happiness, sadness, and calmness, but not for agitation. Third, musical features should significantly influence both the accuracy of emotion judgements and the perceived emotional intensity, with the strength and relevance of these predictors varying by emotion and culture. Together, the three predictions support our overarching purpose: to examine how cultural biases shape emotion perception in response to different musical traditions, particularly unfamiliar musical traditions.

## 2. Methods

### 2.1. Participants

One hundred Western (White-European) participants from the UK, USA, New Zealand, and Australia were recruited through Prolific and completed the online experiment in exchange for £4 for approximately 20 min of participation. All participants met the inclusion criteria: identifying as White/Caucasian, enjoyed listening to music, and had no or limited exposure to Chinese music. Ethics approval was granted by the Macquarie University Human Research Ethics Committee (No: 520231435649832, 2 May 2023).

Participants ranged in age from 20 to 75 years (*M* = 41.58, *SD* = 14.70) and 48 were females. Most participants were non-musicians with minimal formal instrumental training (*M* = 1.55, *SD* = 3.17). Specifically, 36% self-identified as non-musicians, 46% as music-loving non-musicians, 12% as amateur musicians, and 6% as serious amateur musicians.

An a priori power analysis recommended 54 participants for paired-samples *t*-test or analysis of variance (ANOVA) and 107 participants for regression analysis to achieve a medium effect size at 95% power [37]. Therefore, our study included 100 participants, which aligned with the median sample size in cross-cultural emotion recognition research [38].

### 2.2. Music Stimuli

Music from Western classical violin and Chinese traditional *erhu* bowed instrumental genres was selected in a pilot study and validated by cultural experts for emotional connotations and cultural origins [36,39,40]. Table 1 presents the means and standard deviations of emotion ratings and confidence levels in identifying the music’s cultural origin by expert musicians, grouped by emotion type. Bolded values indicate the highest-rated emotion that matched the intended target (e.g., happiness ratings for music intended to convey happiness), and confirms that the excerpts conveyed the intended emotions suitable for this study. The final selection included 24 ten-second music excerpts for each culture, with six excerpts per emotion type (happiness, sadness, agitation, and calmness). Downloadable music excerpts are available from the Open Science Framework (https://osf.io/3whjz/). Further details, including the purpose, participants, music selection, procedure, and a summary of validated stimuli, are available in Supplementary Materials.

### 2.3. Procedure

Participants completed this experiment via Qualtrics, hosted through the Prolific platform. Upon entry, they provided informed consent, Prolific ID, and ethnicity for pre-screening. Prior to the main the experiment, participants were informed that they would listen to 48 music excerpts, each lasting ten seconds, equally divided between Western and Chinese music. To ensure familiarity with the experimental procedure, two practice trials were administered.

The main experiment comprised three sequential phases for each music excerpt. First, participants listened to each music excerpt once and were asked to classify its cultural origin (Western or Chinese music) using a forced-choice question, and to respond as quickly and accurately as possible. The playback stopped immediately once the response was made. Second, participants rated their familiarity with and enjoyment of the music. Third, participants rated the perceived intensity of happiness, sadness, agitation, and calmness for each excerpt using a modified forced-choice paradigm (only one emotion per excerpt could be assigned the highest intensity rating).

The order of excerpts was randomised within the experiment, and five attention-check questions were interspersed throughout the task. Upon completion of all 48 trials, participants answered a brief demographic questionnaire, including musician status, measured using a single-item scale (1 = Non-musician to 6 = Professional musician) from [41].

### 2.4. Design and Analysis

This study adopted a 2 × 4 within-subjects design with two independent variables: (1) musical culture (Western, Chinese) and (2) intended emotion (happiness, sadness, agitation, calmness). Dependent variables fell into three categories: (1) ratings of familiarity, enjoyment, and perceived intensity of four emotions; (2) accuracy in recognising the intended emotion; and (3) acoustic features extracted from the music stimuli. Data from the cultural classification judgements are reported separately [26].

All ratings were measured using seven-point Likert scales (1 represented “not at all”, 4 “moderate”, and 7 “very much”). Recognition accuracy was calculated based on the proportion of trials where the highest-rated emotion matched the intended emotion, as described in [42]. A score of “1” was assigned for correct matches (e.g., the highest happy rating for music-conveyed happiness) and “0” for incorrect matches. Recognition accuracy was then expressed as a percentage. We also calculated the unbiased hit rate (*H_u_*), which considered response bias as outlined in [43]. Additionally, we calculated the probability of chance rate (*P_c_*) and both *H_u_* and *P_c_* were normalised using the arcsine transformation. Significant differences between *H_u_* and *P_c_* indicated accuracy at a level above chance. We conducted a series of pairwise comparisons between Western and Chinese music cultures within each dependent variable.

To examine acoustic predictors of emotion ratings and recognition accuracy, we conducted stepwise multiple linear regression analyses using features extracted with MIR Toolbox 1.8.2 [44] in MATLAB (version R2023b). A total of 20 features were extracted across six categories, following the framework in [13], including (1) dynamics (RMS, low energy); (2) rhythm (attack time, tempo, pulse clarity, event density); (3) timbre (spectral centroid, spectral irregularity, spectral entropy, roughness, MFCC 2, spectral flux, brightness); (4) register (salient pitch); (5) tonality (key clarity and mode); and (6) musical novelty (spectral novelty, rhythm novelty, tonal novelty, register novelty). All statistical analyses were conducted using SPSS v.28.

### 2.5. Test of Assumptions and Possible Covariates

Using the *z*-score cut-off criterion of ±3.29 [45], no statistical outliers were detected. Some violations of normality were found, but they were not substantial and no action was taken. A 2 (culture) × 4 (intended emotion) × 2 (gender) mixed ANOVA was conducted to explore gender as a factor influencing emotional intensity. There were no significant interaction effects of gender and culture (*p* = 0.244), nor of gender and emotional intensity (*p* = 0.375). Pearson correlations between age and familiarity ratings were non-significant for both Western classical music (*r* = −0.09, *p* = 0.399) and Chinese music (*r* = −0.17, *p* = 0.089). Therefore, gender and age were not included in the main analyses.

## 3. Results

Western listeners rated Western classical music as significantly more familiar (*M* = 3.34, *SE* = 0.15) than Chinese music (*M* = 2.25, *SE* = 0.11), *t*(99) = 11.34, *p* < 0.001, *d* = 1.13. Similarly, they reported Western music as more enjoyable (*M* = 3.60, *SE* = 0.13) than Chinese music (*M* = 3.28, *SE* = 0.12), *t*(99) = 3.14, *p* = 0.002, *d* = 0.31.

### 3.1. Emotion Recognition Accuracy Across Cultures

Table 2 presents descriptive statistics of emotion recognition accuracy for happiness, sadness, agitation, and calmness in response to Western classical and Chinese music. Accuracy was measured using two methods: the proportion of trials where the highest-rated emotion matched the intended emotion (%) and the unbiased hit rate (*H_u_*). Results comparing the chance rate (*P_c_*) with the unbiased accuracy rate (*H_u_*) for each emotion type revealed that all *H_u_* rates were significantly greater than chance rates, *t*s(99) ≥ 8.90, *p-*values < 0.001, *d*s > 0.89, indicating above-chance accuracy in recognising each emotion type within both Western classical and Chinese music. This suggests that Western listeners could successfully decode emotions conveyed in both musical traditions.

Next, we compared recognition accuracy using the proportion rate (%) and the unbiased hit rate (*H*_u_) for each emotion across Western and Chinese music. The results revealed that Western music conveying happiness was recognised significantly more accurately than Chinese music conveying happiness, as measured by the proportion rate, *t*(99) = 3.72, *p* < 0.001, *d* = 0.37. The mean difference in the unbiased hit rate (*M* = 0.21, *SE* = 0.03) was also significant, *t*(99) = 7.36, *p* < 0.001, *d* = 0.74, indicating that happiness was recognised more accurately in Western music than in Chinese music.

Western music conveying sadness was recognised significantly more accurately than Chinese music conveying sadness, as measured by the proportion rate, *t*(99) = 5.56, *p* < 0.001, *d* = 0.56. However, the mean difference in the unbiased hit rate (*M* = 0.01, *SE* = 0.03) was not significant, *t*(99) = 0.44, *p* = 0.661, *d* = 0.04, indicating sadness was recognised similarly in Western and Chinese music when unbiased accuracy was analysed.

Chinese music conveying agitation was recognised significantly more accurately than Western music conveying agitation, as measured by the proportion rate, *t*(99) = 5.32, *p* < 0.001, *d* = 0.53. The mean difference in the unbiased hit rate (*M* = 0.07, *SE* = 0.03) was also significant, *t*(99) = 2.56, *p* = 0.012, *d* = 0.26, indicating that agitation was recognised more accurately in Chinese music than in familiar Western classical music.

There was no significant difference in recognition accuracy for calmness between Western music, as measured by the proportion rate, *t*(99) = −1.96, *p* = 0.052, *d* = −0.20. The mean difference in the unbiased hit rate (*M* = −0.03, *SE* = 0.02) was also not significant, *t*(99) = −1.70, *p* = 0.093, *d* = −0.17. This suggests that calmness was recognised similarly for Western and Chinese music.

### 3.2. Perceived Emotional Intensity Across Cultures

Table 3 presents a matrix of music stimuli and mean emotional intensity ratings for each emotion type in Western classical and Chinese music. The highest mean intensity rating for each perceived emotion that matched the emotion conveyed by the music is highlighted in bold. These intended emotions were validated by expert musicians during the pilot study (see Table 1).

We compared ratings of matched emotional intensity (e.g., ratings of happiness for music-conveyed happiness) between Western classical and Chinese music. Western music conveying happiness was rated significantly happier than Chinese music conveying happiness, *t*(99) = 4.09, *p* < 0.001, *d* = 0.41. Western music conveying sadness was rated significantly sadder than Chinese music conveying sadness, *t*(99) = 8.05, *p* < 0.001, *d* = 0.81. Chinese music conveying agitation was rated significantly more agitated than Western music conveying agitation, *t*(99) = 5.27, *p* < 0.001, *d* = 0.53. No significant difference was found in calmness ratings between Western and Chinese music conveying calmness, *t*(99) = 0.15, *p* = 0.884, *d* = 0.02.

**Figure 1:**
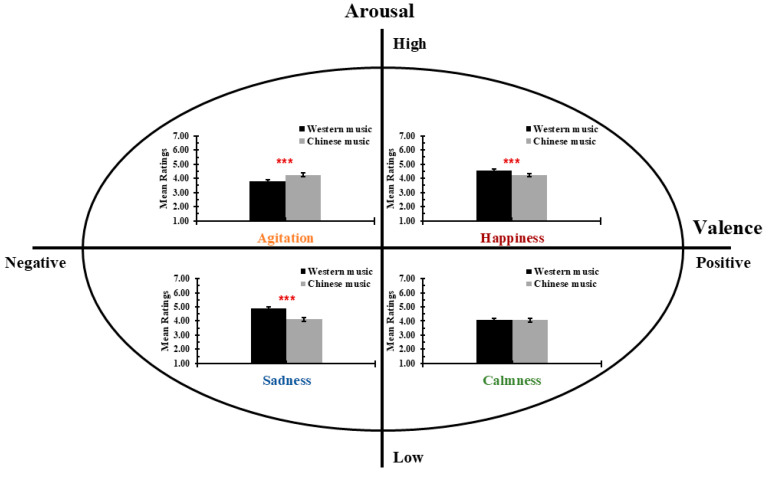
Comparison of perceived intensity of emotions in music across Western classical and Chinese music. The figure illustrates the perceived intensity ratings for four emotions—agitation, happiness, sadness, and calmness—in Western classical and Chinese music, mapped onto the arousal–valence circumplex model [20]. Agitation (high arousal, negative valence) was perceived as more intense in Chinese music, while happiness (high arousal, positive valence) and sadness (low arousal, negative valence) were perceived more intense in Western music. No significant difference was found for calmness (low arousal, positive valence). Ratings were on a seven-point Likert scale. Error bars denote the standard error of the mean. *** *p* < 0.001.

As illustrated in Figure 1, perceived emotional intensity varied across cultures, with greater intensity ratings for happiness and sadness in Western classical music and greater ratings for agitation in Chinese music. There was no significant difference in calmness between the two music cultures. These findings suggest that the amplification of emotional intensity differed between cultures based on emotional qualities that align with arousal and valence characteristics.

### 3.3. Musical Features Predicting Emotion Perception

We conducted stepwise multiple linear regression analyses to examine whether musical features predicted emotion recognition and perceived emotion intensity. This method is commonly used to examine the roles of musical features in affective judgement [13,22]. The results presented here include only statistically significant predictors. The full set of analyses are provided in Supplementary Materials (Tables S5–S13). Stepwise methods excluded six features for lack of significant contribution. The remaining specific features were classified into the six general categories: dynamics, rhythm, timbre, register, tonality, and musical novelty. Some acoustic differences between Chinese and Western music were observed in these categories, with statistical data available in Supplementary Materials (Table S5).

#### 3.3.1. Predictors of Perceived Emotion Recognition Accuracy

For Western music conveying happiness, a model with one acoustic predictor explained 17% of the variance in recognition accuracy, *F*(1, 22) = 5.79, *R*^2^_adj._ = 0.17, *p* = 0.025, *f*^2^ = 0.26. Event density (a rhythmic feature) was a positive predictor. For Chinese music conveying happiness, a model with three acoustic predictors explained 61% of the variance in recognition accuracy, *F*(3, 20) = 12.87, *R*^2^_adj._ = 0.61, *p* < 0.001, *f*^2^ = 1.93. Tonal and spectral novelties (both features of musical novelty) and roughness (a timbral feature) were positive predictors.

For Western music conveying sadness, a model with four acoustic predictors explained 66% of the variance in recognition accuracy, *F*(4, 19) = 12.26, *R*^2^_adj._ = 0.66, *p* < 0.001, *f*^2^ = 1.96. Pulse clarity (a rhythmic feature), key clarity (a tonal feature), and spectral entropy (a timbral feature) were negative predictors, while tempo was a positive predictor. For Chinese music conveying sadness, a model with three acoustic predictors explained 78% of the variance in recognition accuracy, *F*(3, 20) = 27.87, *R*^2^_adj._ = 0.78, *p* < 0.001, *f*^2^ = 3.50. Roughness and spectral flux (both timbral features) and tonal novelty (a musical novelty feature) were negative predictors.

No significant model was found for Western music conveying agitation. For Chinese music conveying agitation, a model with four acoustic predictors explained 94% of the variance in recognition accuracy, *F*(4, 19) = 95.32, *R*^2^_adj._ = 0.94, *p* < 0.001, *f*^2^ = 16.54. Spectral flux (a timbral feature) and pulse clarity (a rhythmic feature) were positive predictors, while RMS (a dynamic feature) and spectral novelty (a feature of musical novelty) were negative predictors.

For Western music conveying calmness, a model with two acoustic predictors explained 30% of the variance in recognition accuracy, *F*(2, 21) = 6.00, *R*^2^_adj._ = 0.30, *p* = 0.009, *f*^2^ = 0.57. Spectral entropy (a timbral feature) was a negative predictor, while attack time (a rhythmic feature) was a positive predictor. For Chinese music conveying calmness, a model with two acoustic predictors explained 64% of the variance in recognition accuracy, *F*(2, 21) = 21.37, *R*^2^_adj._ = 0.64, *p* < 0.001, *f*^2^ = 1.77. Spectral flux (a timbral feature) was a negative predictor, while RMS (a feature of dynamics) was a positive predictor.

**Figure 2:**
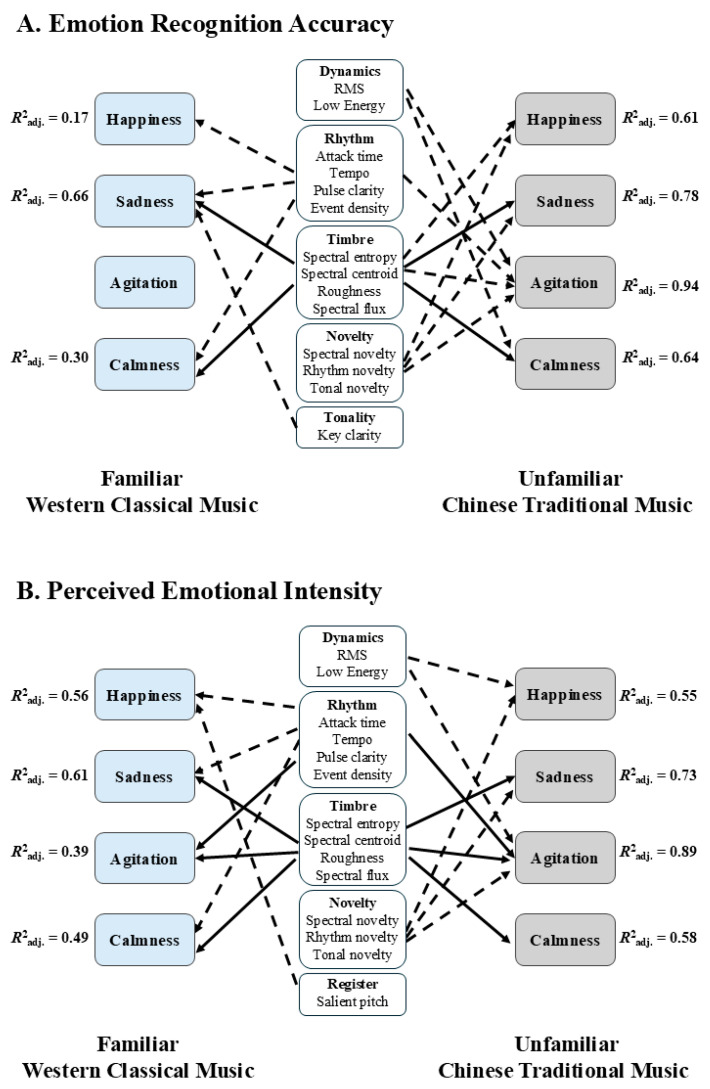
Musical features as predictors of emotion perception. This figure illustrates significant predictors of emotion perception: Panel (**A**) represents emotion recognition accuracy, and Panel (**B**) represents perceived emotional intensity. Solid black arrows represent shared predictors between Western classical and Chinese traditional music, while dashed black arrows indicate predictors specific to each music tradition. Variance explained (adjusted *R*^2^) is displayed next to each model.

Figure 2A illustrates shared and culture-specific predictors of emotion recognition accuracy across Western and Chinese music. Shared predictors are defined as musical features that significantly predict the same perceived emotion in both traditions. In contrast, culture-specific predictors are significant only in one tradition. Our results revealed that timbral features predicted sadness and calmness in both traditions, as well as happiness and agitation in Chinese music only. Rhythmic features predicted happiness, sadness, and calmness in Western music and agitation in Chinese music. Dynamics predicted agitation and calmness, while musical novelty predicted happiness, sadness, and agitation only in Chinese music. Tonal novelty predicted sadness only in Western music.

#### 3.3.2. Predictors of Perceived Emotional Intensity

For the intensity of perceived happiness in response to Western music, a model with three acoustic predictors explained 56% of the variance in intensity, *F*(3, 20) = 10.79, *R*^2^_adj._ = 0.56, *p* < 0.001, *f*^2^ = 1.36. Pulse clarity and salient pitch were positive predictors, while tempo was a negative predictor. In Chinese music, a model with two acoustic predictors explained 55% of the variance in intensity, *F*(2, 21) = 15.26, *R*^2^_adj._ = 0.55, *p* < 0.001, *f*^2^ = 1.45. Tonal novelty was a positive predictor, while low energy (a feature of dynamics) was a negative predictor.

For Western music conveying sadness, a model with three acoustic predictors explained 61% of the variance in intensity, *F*(3, 20) = 12.91, *R*^2^_adj._ = 0.61, *p* < 0.001, *f*^2^ = 1.93. Pulse clarity and spectral centroid were negative predictors, while tempo was a positive predictor. In Chinese music, a model with two acoustic predictors explained 73% of the variance in intensity, *F*(2, 21) = 31.34, *R*^2^_adj._ = 0.73, *p* < 0.001, *f*^2^ = 2.98. Roughness and tonal novelty were negative predictors.

For Western music conveying agitation, a model with two acoustic predictors explained 39% of the variance in intensity, *F*(2, 21) = 8.29, *R*^2^_adj._ = 0.39, *p* = 0.002, *f*^2^ = 0.79. Spectral entropy was a positive predictor, while attack time (a feature of rhythm) was a negative predictor. In Chinese music, a model with five acoustic predictors explained 89% of the variance in intensity, *F*(5, 18) = 38.58, *R*^2^_adj._ = 0.89, *p* < 0.001, *f*^2^ = 10.76. Spectral flux, low energy, and event density were positive predictors, while RMS and rhythm novelty (a feature of musical novelty) were negative predictors.

For Western music conveying calmness, a model with two acoustic predictors explained 49% of the variance in intensity, *F*(2, 21) = 11.85, *R*^2^_adj._ = 0.49, *p* < 0.001, *f*^2^ = 1.13. Spectral entropy was a negative predictor, while attack time was a positive predictor. In Chinese music, a model with two acoustic predictors explained 58% of the variance in intensity, *F*(2, 21) = 16.73, *R*^2^_adj._ = 0.58, *p* < 0.001, *f*^2^ = 1.59. Spectral flux and spectral entropy were negative predictors.

Figure 2B, illustrates shared and culture-specific predictors of perceived emotional intensity in Western and Chinese music. Timbral features predicted sadness, agitation, and calmness in both cultures, while rhythmic features predicted agitation in both but also happiness and sadness in Western music. Additionally, pitch register predicted happiness only in Western music. Dynamics predicted happiness and agitation, whereas musical novelty predicted happiness, sadness, and agitation exclusively in Chinese music.

## 4. Discussion

This study investigated cultural biases in emotion perception by examining Western listeners’ responses to Western classical and Chinese traditional music. Compared with Chinese traditional music, Western classical music was perceived as more familiar and enjoyable. Happiness was more accurately recognised and perceived as more intense in Western classical music, whereas agitation was more accurately recognised and perceived as more intense in Chinese music. Sadness was also perceived as more intense in Western music.

Although listeners judged categorical emotions, response biases varied depending on the valence and arousal of the emotion judged. Recognition data varied across cultures based on valence: the positive emotion of happiness was more accurately recognised in Western music, whereas the negative emotion of agitation was better recognised in Chinese music. Emotional intensity data varied across cultures: happiness and sadness were perceived as more intense in Western music whereas agitation was perceived as more intense in Chinese music. These findings suggest that cultural familiarity and emotional characteristics shape perceptual biases in cross-cultural music perception.

Musical features predicted emotional judgements. Timbral features predicted recognition accuracy and the intensity of sadness and calmness in both Western and Chinese music. Timbral and rhythmic features also predicted the intensity of agitation across cultures, but they did not predict its recognition in Western music. For Chinese music only, dynamics, rhythmic features and musical novelty predicted recognition accuracy for agitation.

### 4.1. Cultural Familiarity and Emotion Perception

Greater familiarity with and enjoyment of Western classical music can be explained by Western listeners’ enculturation and prior exposure [2,13]. Long-term exposure to Western music provided access to the regularities of this musical system, enabling the utilisation of both psychophysical (culture-general) and culture-specific cues when making judgements. The dual availability of these cues in Western music facilitated emotional interpretation, enhancing recognition accuracy and perceived emotional intensity.

However, whereas familiarity with Western music may have improved recognition accuracy and perceived intensity of happiness, a lack of familiarity with Chinese music may have led to greater recognition accuracy and perceived intensity of agitation. A lack of familiarity with Chinese music may have amplified the emotion of agitation, likely because unpredictable structures create a sense of tension.

For sadness, familiarity with Western music heightened emotional intensity but did not improve recognition accuracy. For calmness, no in-culture advantage was observed, possibly because the emotion of calmness is expressed similarly across cultures. Overall, these findings highlight that cultural biases are shaped by key processes in music perception, such as long-term exposure, expectations, and emotional appraisal [2,34,35].

### 4.2. Musical Features and Emotion Perception

Agitation was judged somewhat differently in Western and Chinese music. Specifically, no musical cues predicted the recognition of agitation in Western classical music, suggesting that listeners did not rely on low-level features when interpreting agitation in familiar music. In contrast, multiple musical cues predicted agitation in Chinese music: dynamics, rhythmic features, timbral features, and musical novelty. This finding suggests that Western listeners drew from a large set of low-level musical cues in order to judge the emotion of agitation in Chinese music, possibly because they were unfamiliar with this style of music. For perceived intensity, timbral and rhythmic features were consistent predictors of agitation across cultures. In Chinese music, dynamics and musical novelty were also associated with emotional intensity, acting as emotional amplifiers for unfamiliar music. These findings are consistent with previous research indicating that arousal responses to music are driven by low-level acoustic cues [46,47]. This study further demonstrates that specific acoustic cues associated with high-arousal negative emotions likely contribute to cross-cultural biases in music perception.

Beyond agitation, timbral features also emerged as shared predictors of judgements of sadness and calmness, aligning with research on timbre’s fundamental role in emotion perception across Western and Chinese music [47]. Happiness, in contrast, showed minimal reliance on acoustic cues, supporting the universal recognition of basic emotions [23].

Why were some features such as those associated with timbre identified as shared predictors of emotion, while others, like dynamics, we identified as culture-specific predictors? One possibility is that timbre influences emotional responses through qualities linked to core auditory perception and affective systems, which are rooted in biologically and evolutionarily shaped mechanisms [35]. In our findings, spectral entropy—a timbral feature associated with complexity—negatively predicted calmness in both cultures. This finding suggests that lower spectral entropy reflects a more stable and soothing sound that is associated with a sense of calmness, regardless of the music’s cultural origin. In contrast, dynamics—defined here as the overall dynamic contour of a musical performance—may reflect culturally learned expressive norms, and its emotional interpretation may vary across traditions.

Our findings align with theoretical models of music perception [16,22,34,35]. When encountering unfamiliar music, listeners rely on psychophysical cues to judge emotional intentions, particularly for complex emotions like agitation. When encountering familiar music, listeners can incorporate culture-specific cues, such as rhythmic structure in Western music and dynamics in Chinese music, to refine interpretation. That is, cultural familiarity shapes cue utilisation. Moreover, cue flexibility allows listeners to adaptively adjust reliance on available cues based on cultural familiarity. When familiar cues are absent in Chinese music, unfamiliar attributes such as roughness and dissonance [48] are difficult to interpret or predict, leading to persistent violations of expectancies. Violations of expectancies, in turn, amplify the perception of agitation and give rise to a cross-cultural bias in judgements. These cue-based explanations underscore the way listeners flexibly integrate culturally shared and culture-specific cues, dynamically adjusting cue usage based on familiarity to interpret emotions in cross-cultural contexts.

This research provides novel insight into the processes and factors underlying cultural biases in emotional judgements of music, demonstrating how cultural familiarity, musical features, and emotional qualities interact to shape these biases. Given that such biases likely reflect broader cultural tendencies, active engagement with unfamiliar music could foster intercultural empathy. Indeed, research suggests that rich active engagement with unfamiliar musical traditions can increase familiarity and reduce cultural bias [49,50]. In educational and therapeutic settings, thoughtful engagement with cross-cultural music may encourage openness to the values and aesthetics of other cultures.

Building on this foundation, future studies could assess whether similar patterns of cross-cultural bias are observed in listeners from non-English Western and non-Western countries, such as Chinese listeners judging both Western classical and Chinese traditional music. Further research might also explore other musical genres—including Hindustani music or Middle Eastern traditions like Arabic maqam, Persian classical music, or Turkish makam—and extend investigations to include additional emotions, such as anger. Such work would deepen our understanding of the dynamics of cross-cultural emotional biases in music perception.

Furthermore, understanding how the brain processes culturally familiar and unfamiliar music is important because differences in activation and connectivity may account for biases in music perception, including the persistence of such biases and a difficulty in resisting them. Listening to culturally familiar or unfamiliar (foreign) music is hypothesised to engage differing brain areas at each processing stage [51,52]. Early auditory processing occurs in the primary auditory cortex (A1) and superior temporal gyrus (STG), where sound features like pitch and timbre are analysed [51]. The classification of music as familiar or foreign might involve the planum temporale and angular gyrus, which may help differentiate culturally relevant patterns and integrate semantic knowledge [52]. Emotion recognition likely engages the amygdala, anterior insula, and ventral striatum, processing arousal, emotional salience, and reward [32]. Enjoyment could involve the medial prefrontal cortex (mPFC) and anterior cingulate cortex (ACC), linking personal relevance with emotional–cognitive integration [32,53]. Finally, the temporoparietal junction (TPJ) and posterior superior temporal sulcus (pSTS) may be involved in interpreting cultural associations [54]. Differences in activation and connectivity within these regions may underlie many of the biases that favour culturally familiar music. One specific avenue for future research is to investigate whether the heightened activation of neural networks associated with threat and novelty processing, such as the amygdala and anterior insula, underpins the cultural bias in perception of high-arousal negative emotions (such as agitation, fear, and anger) in unfamiliar music, similar to those observed for intergroup biases [55,56].

## 5. Conclusions

This study investigated how cultural biases in emotion perception manifest when Western listeners engage with familiar music from their own culture and unfamiliar music from another culture. Cultural biases were observed in both emotion recognition and perceived emotional intensity. These biases are shaped by cultural familiarity, vary depending on the emotion, and are influenced by musical features. By including the understudied emotion of agitation, the study expands the palate of emotions investigated in cross-cultural music research. Cultural biases in music perception may reflect broader societal biases, and the study of them offers a valuable model for understanding how biases can impact upon interactions in other aspects of life.

## Figures and Tables

**Table 1:** Matrix of music stimuli and mean emotion ratings by cultural expert musicians.

Culture	Happiness	Sadness	Agitation	Calmness	Cultural Recognition
Western music					
Happiness	**6.25 (0.25)**	1.58 (0.25)	2.63 (0.56)	3.90 (0.58)	6.88 (0.00)
Sadness	1.81 (0.32)	**5.71 (0.53)**	2.58 (1.07)	3.60 (0.77)	6.88 (0.00)
Agitation	2.63 (0.99)	3.48 (1.12)	**5.65 (0.74)**	1.69 (0.49)	6.79 (0.13)
Calmness	4.04 (0.53)	2.85 (0.8)	1.77 (0.41)	**5.58 (0.52)**	6.88 (0.00)
Chinese music					
Happiness	**6.08 (0.32)**	1.31 (0.21)	3.25 (0.97)	3.35 (1.18)	6.77 (0.29)
Sadness	2.06 (0.30)	**5.46 (0.69)**	2.81 (0.74)	3.50 (0.75)	6.44 (0.47)
Agitation	3.52 (0.24)	2.21 (0.44)	**5.96 (0.52)**	1.56 (0.39)	6.08 (0.38)
Calmness	4.10 (1.09)	2.75 (1.2)	1.96 (0.51)	**5.44 (0.29)**	6.69 (0.19)

Note. Standard deviations are presented in parentheses. Western music classical and Chinese music excerpts (24 per culture, 6 per emotion type) were rated by Western and Chinese expert musicians (*N* = 8 each). All ratings were on a seven-point scale (1 = “Not at all”, 4 = “Moderately”, and 7 = “Very much”).

**Table 2:** Descriptive statistics for emotion recognition accuracy across music cultures.

Emotion	Western Music		Chinese Music	
	%	*H_u_*	%	*H_u_*
Happiness	63.33 (2.83)	42.96 (2.26)	53.33 (2.68)	25.31 (1.52)
Sadness	67.00 (2.93)	35.41 (1.96)	50.17 (3.27)	33.35 (2.32)
Agitation	45.33 (2.76)	36.37 (2.42)	56.67 (3.07)	42.63 (2.61)
Calmness	38.67 (2.78)	18.59 (1.46)	44.67 (3.01)	21.76 (1.74)

Note. Mean and standard error (in parentheses) were reported. % = proportion of correct response rate; *H_u_* = unbiased hit rate.

**Table 3:** Matrix of mean perceived emotional intensity ratings for each intended emotion (*N* = 100).

Intended Emotions	Emotional Intensity Ratings			
	Happiness	Sadness	Agitation	Calmness
Western music				
Happiness	**4.54 (0.13)**	1.96 (0.08)	2.19 (0.11)	3.37 (0.12)
Sadness	2.01 (0.08)	**4.89 (0.14)**	2.13 (0.10)	3.56 (0.11)
Agitation	2.78 (0.09)	3.03 (0.11)	**3.78 (0.15)**	2.29 (0.10)
Calmness	2.88 (0.10)	3.92 (0.12)	1.80 (0.08)	**4.08 (0.12)**
Chinese music				
Happiness	**4.22 (0.12)**	2.22 (0.10)	2.48 (0.10)	3.07 (0.11)
Sadness	2.36 (0.09)	**4.10 (0.14)**	2.03 (0.10)	3.75 (0.13)
Agitation	3.61 (0.12)	1.96 (0.08)	**4.25 (0.15)**	1.81 (0.08)
Calmness	3.45 (0.11)	3.00 (0.11)	1.81 (0.08)	**4.06 (0.12)**

Note. Bolded values indicate the highest emotional intensity for each intended emotion. Ratings were measured on a seven-point Likert scale, with mean values and standard errors (in parentheses) reported.

## Data Availability

The original contributions presented in this study are included in the article/Supplementary Materials. Downloadable music excerpts are available from the Open Science Framework (https://osf.io/3whjz/). Further inquiries can be directed to the corresponding author.

## Supplementary Materials

The following supporting information can be downloaded at: https://www.mdpi.com/article/10.3390/brainsci15050477/s1, Supplementary Method: Music Stimuli Pilot Study, Table S1: Western Music Stimulus and Emotion Ratings by Western Expert Musicians; Table S2: Chinese Music Stimulus and Emotion Ratings by Chinese Expert Musicians; Table S3: Western Music Stimuli Source Tracks; Table S4: Chinese Music Stimuli Source Tracks. Supplementary Results: Musical Feature Analysis; Table S5: Comparing Musical Features; Table S6: Musical Features Predicting Happiness Accuracy; Table S7: Musical Features Predicting Sadness Accuracy; Table S8: Musical Features Predicting Agitation Accuracy; Table S9: Musical Features Predicting Calmness Accuracy; Table S10: Musical Features Predicting Happiness Intensity; Table S11: Musical Features Predicting Sadness Intensity; Table S12: Musical Features Predicting Agitation Intensity; Table S13: Musical Features Predicting Calmness Intensity; References [13,24,36,39,40,44] are cited in the Supplementary Material.
