# Supplementary material for: Cross-Cultural Biases of Emotion Perception in Music
Source: Brain Sci. 2025 Apr 29;15(5):477. doi: 10.3390/brainsci15050477 (PMC12110013; doi:10.3390/brainsci15050477)
Supplement: Supplementary file 1 [file brainsci-15-00477-s001.zip › brainsci-3590855-supplementary.pdf]

Cross-Cultural Biases of Emotion Perception in Music

Supplementary Method

Music Stimuli: Pilot Study

The purpose of the pilot study was to confirm that the music stimuli selected for the main Experiment conveyed the intended emotional connotations of agitation, calmness, happiness, and sadness in both Western and Chinese music. Furthermore, the study ensured that the music stimuli were appropriately identified as either Western or Chinese music.

Participants

Eight experts in Western music validated the Western classical music stimuli and eight experts in Chinese music validated the Chinese traditional music stimuli. These musicians were required to have over 10 years of experience studying and/or performing on their musical instruments. This criterion for selecting musicians was similar to that used in previous research [1].

Music Stimuli Selection

We selected music involving bowed string instruments from both Western and Chinese cultures such that the class of instrument in the two cultural genres were matched [2]. Western stimuli included 26 classical viola/violin music by 17 composers, spanning from the baroque to late romantic periods. Chinese stimuli included 13 pieces of Chinese classical/traditional erhu/jinhu music by various traditional composers. A mix of solo and ensemble pieces where a target instrument was featured were sourced from iTunes.

Using Audacity version 3.3.2, 10-second excerpts from these pieces were created with the addition of 500ms of silence at the beginning of each file and 500ms of fade out at the end. The stimuli were designed to convey four emotions representing the four quadrants of the arousal-valence circumplex model: happiness, sadness, agitation, and calmness. These same emotions were used in comparing emotional characteristics between Western and Chinese bowed string instruments [3]. In total, 32 excerpts for each music culture were

selected, with eight per emotion category.

Procedure

Western musicians completed the Qualtrics survey containing Western music stimuli, while Chinese musicians completed the survey containing Chinese music stimuli, with the option to select instructions in Simplified Chinese. The expert musicians were asked to rate the four emotional connotations on a 7-point Likert scale (1 indicates “Not at all,” 4 indicates “Moderately,” and 7 indicates “Very much”). Recognising that music often conveys mixed emotions, they were asked to assign the highest rating to a single emotion that they perceived as the most salient in each excerpt. After that, they were asked to rate their level of agreement with the classification of the music as Western (in the Western music survey) or Chinese (in the Chinese music survey) to confirm its cultural origin, using a 7-point Likert scale (1 indicates “Strongly disagree,” 4 indicates “Unsure,” and 7 indicates “Strongly agree”).

Initial validation indicated a need for additional excerpts for Chinese music, as some excerpts had highest ratings not matching the intended emotion. Consequently, eight agitated and four calming excerpts from a validated dataset by Xie and Gao [4] were also assessed by the same Chinese experts.

Summary of Validated Music Stimuli

Tables S1 and S2 display the mean ratings for each excerpt of Western classical and Chinese music. As shown in bold text, the highest ratings of perceived emotional connotations aligned with the intended emotions for both cultures and were accompanied by high confidence in cultural recognition, thereby validating that the excerpts conveyed the intended emotions appropriate for this study. Tables S3 and S4 provide the source for each of the final excerpts chosen for the study. Downloadable music excerpts are available from https://osf.io/3whjz/.

Table S1

Each Western Music Stimulus and Emotion Ratings by Western Expert Musicians (N=8)

Western	Happiness	Sadness	Agitation	Calmness	Confidence
VWEx1	2.88	3.38	**4.88**	1.63	6.63
VWEx2	2.00	3.63	**5.75**	1.50	6.88
VWEx3	4.38	2.88	**5.75**	1.50	6.63
VWEx4	1.88	1.88	**6.88**	1.00	6.88
VWEx5	1.75	5.25	**5.75**	2.38	6.88
VWEx8	2.88	3.88	**4.88**	2.13	6.88
**Agitated**	2.63 (0.99)	3.48 (1.12)	**5.65 (0.74)**	1.69 (0.49)	6.79 (0.13)
VWEx9	3.25	3.75	1.38	**6.00**	6.88
VWEx10	4.38	3.38	1.38	**6.25**	6.88
VWEx12	3.50	2.38	2.38	**5.00**	6.88
VWEx13	4.38	1.75	2.13	**5.00**	6.88
VWEx14	4.50	2.38	1.63	**5.75**	6.88
VWEx16	4.25	3.50	1.75	**5.50**	6.88
**Calm**	4.04 (0.53)	2.85 (0.8)	1.77 (0.41)	**5.58 (0.52)**	6.88 (0)
VWEx17	**6.13**	1.50	1.88	4.75	6.88
VWEx18	**6.13**	1.38	2.75	3.75	6.88
VWEx19	**6.50**	1.75	2.88	3.75	6.88
VWEx21	**6.63**	1.38	3.00	3.88	6.88
VWEx23	**6.13**	2.00	2.00	4.25	6.88
VWEx24	**6.00**	1.50	3.25	3.00	6.88
**Happy**	**6.25 (0.25)**	1.58 (0.25)	2.63 (0.56)	3.9 (0.58)	6.88 (0)
VWEx25	1.75	**6.25**	1.38	4.50	6.88
VWEx27	2.00	**5.13**	1.50	4.25	6.88
VWEx28	1.63	**5.50**	3.00	2.75	6.88
VWEx30	1.50	**6.25**	3.63	3.00	6.88
VWEx31	2.38	**5.13**	3.88	3.00	6.88
VWEx32	1.63	**6.00**	2.13	4.13	6.88
**Sad**	1.81 (0.32)	**5.71 (0.53)**	2.58 (1.07)	3.6 (0.77)	6.88 (0)

Table S2

Each Chinese Music Stimulus and Emotion Ratings by Chinese Expert Musicians (N=8)

Chinese	Happiness	Sadness	Agitation	Calmness	Confidence
VCEx7	3.50	2.50	**6.25**	1.25	6.50
VCEx33	3.25	2.00	**6.50**	1.38	5.50
VCEx36	3.63	1.63	**6.38**	1.38	6.00
VCEx37	3.25	2.75	**5.75**	1.25	6.00
VCEx43	3.88	1.88	**5.75**	2.00	6.00
VCEx44	3.63	2.50	**5.13**	2.13	6.50
**Agitated**	3.52 (0.24)	2.21 (0.44)	**5.96 (0.52)**	1.56 (0.39)	6.08 (0.38)
VCEx13	5.13	1.63	2.13	**5.38**	6.75
VCEx14	5.38	1.88	2.13	**5.63**	6.75
VCEx15	3.13	4.00	1.88	**5.13**	6.63
VCEx40	3.00	3.88	2.75	**5.13**	6.50
VCEx41	4.75	1.50	1.63	**5.50**	7.00
VCEx42	3.25	3.63	1.25	**5.88**	6.50
**Calm**	4.1 (1.09)	2.75 (1.2)	1.96 (0.51)	**5.44 (0.29)**	6.69 (0.19)
VCEx17	**6.38**	1.38	2.25	4.00	6.88
VCEx18	**6.13**	1.00	4.38	2.50	7.00
VCEx20	**5.63**	1.38	4.00	2.75	6.63
VCEx21	**6.00**	1.50	3.13	3.63	6.25
VCEx22	**5.88**	1.50	3.75	2.00	6.88
VCEx24	**6.50**	1.13	2.00	5.25	7.00
**Happy**	**6.08 (0.32)**	1.31 (0.21)	3.25 (0.97)	3.35 (1.18)	6.77 (0.29)
VCEx26	1.88	**6.13**	2.75	4.25	6.88
VCEx27	2.25	**5.63**	3.00	3.38	6.88
VCEx28	2.13	**5.63**	2.75	3.25	5.63
VCEx29	1.63	**6.13**	2.13	3.63	6.63
VCEx31	2.50	**4.50**	2.13	4.25	6.38
VCEx32	2.00	**4.75**	4.13	2.25	6.25
**Sad**	2.06 (0.3)	**5.46 (0.69)**	2.81 (0.74)	3.5 (0.75)	6.44 (0.47)

Table S3

Western Music Stimuli Source Tracks

Excerpts	Track	CD
VWEx1	01 Csardas	Csardas (Song of Gypsy)
VWEx2	10 Capriccio for Solo Viola, Op. 55	10 Capriccio for Solo Viola, Op. 55
VWEx3	19 La Ronde des lutins, Op. 25	Golden Oldies – More Favourite Encores
VWEx4	08 Viola Sonata, Op. 25, No. 1_ IV	Hindemith: Solo Viola Sonatas
VWEx5	13 The Four Seasons, Concerto No. 2_2	Violin: Classical Music
VWEx8	2-57 String Quartet Op. 76, No. 2, IV	The Norton Recordings, 10th Ed20060
VWEx9	03 Trois mélodies, Op. 7_ No. 1, Apr	Golden Oldies – More Favourite Encores
VWEx10	07 Le Carnaval des animaux, R. 125_	Golden Oldies – More Favourite Encores
VWEx12	28 Fantasia for Solo Viola in E-Flat	Georg Philipp Telemann: Viola Concertos - Overtures - Fantasias
VWEx13	01 Suite for Viola & Orchestra, Grou	Music for Viola & Chamber Orchestra
VWEx14	02 Divertimento in E flat major, K	Mozart: Divertimento, K. 563
VWEx16	6-12 Violin Concerto In E, Op. 64 - I	The Norton Recordings, 10th Ed20060
VWEx17	01 Divertimento in E flat major, K..	Mozart: Divertimento, K. 563
VWEx18	07 String Trio in B flat major, D. 4	Mozart: Divertimento, K. 563
VWEx19	10 Viola Concerto in G Major, TWV 51_1	Violin: Classical Music
VWEx21	1-15 Allegro	Henry Purcell - Historical Anthology
VWEx23	08 The Four Seasons, Concerto No. 1_1	Violin: Classical Music
VWEx24	08 The Four Seasons, Concerto No. 1_2	Violin: Classical Music
VWEx25	03 Trois mélodies, Op. 7_ No. 1, Apr	Golden Oldies – More Favourite Encores
VWEx27	01 Concerto for Viola & String Orche	Pēteris Vasks: Viola Concerto & Symphony No. 1 "Voices"

Excerpts	Track	CD
VWEx28	02 Concerto for Viola & String Orche	Pēteris Vasks: Viola Concerto & Symphony No. 1 "Voices"
VWEx30	13 The Four Seasons, Concerto No. 2_1	Violin: Classical Music
VWEx31	6-10 Violin Concerto In E, Op. 64 - I	The Norton Recordings, 10th Ed20060
VWEx32	2-62 String Quartet In C-Sharp Minor, Op. 131 First Movement	Norton Recorded Anthology Of Western Music - Volume 2 [Disc 8]

Table S4

Chinese Music Stimuli Source Tracks

Excerpts	Track	CD
VCEx7	Outskirts of Beijing 京郊行	Treasures of Chinese Instrumental Music: Bowed Instruments 中国民族乐器典藏 弓絃乐
VCEx33	《双阕》Double Poems	Xie and Gao (2022) study
VCEx36	《满江红》Amaranthus	Xie and Gao (2022) study
VCEx37	《满江红》Amaranthus	Xie and Gao (2022) study
VCEx43	《我的祖国》My country	Xie and Gao (2022) study
VCEx44	《哪吒令》Nezha Order	Xie and Gao (2022) study
VCEx13	Spring Sceneries of Jiangnan 江南春色	Treasures of Chinese Instrumental Music: Erhu 中国民族乐器典藏 二胡
VCEx14	Spring Sceneries of Jiangnan 江南春色	Treasures of Chinese Instrumental Music: Erhu 中国民族乐器典藏 二胡
VCEx15	Beautiful Evening 良宵	Moon Night (10 Erhu Solo)
VCEx40	《沂蒙山小调》Yimeng Mountain tune	Xie and Gao (2022) study
VCEx41	《浏阳河》Liuyang River	Xie and Gao (2022) study
VCEx42	《月儿弯弯照九州》The Crescent Moon Shines Over Jiuzhou`	Xie and Gao (2022) study
VCEx17	Good News 喜讯	Erhu Solo by Wei Xiao Dong 二胡独奏专辑

Excerpts	Track	CD
VCEx18	Horse Race 赛马	Music By Famous Artists - Erhu 二胡演奏巨星名曲
VCEx20	Going To Market 赶集	Treasures of Chinese Instrumental Music: Erhu 中国民族乐器典藏 二胡
VCEx21	Towards A Brighter Future 光明行	Classical Chinese Music - Ten Best Erhu Melody 中国二胡十大金曲
VCEx22	Outskirts of Beijing 京郊行	Treasures of Chinese Instrumental Music: Bowed Instruments 中国民族乐器典藏 弓絃乐
VCEx24	Jasmine Flower 茉莉花	chinesemusicworld-Erhu
VCEx26	Moonlight Reflected On the Er-Quan Spring 二泉映月	Classical Chinese Music - Ten Best Erhu Melody 中国二胡十大金曲
VCEx27	Moonlight Reflected On the Er-Quan Spring 二泉映月	Classical Chinese Music - Ten Best Erhu Melody 中国二胡十大金曲
VCEx28	Song of Melancholy 悲歌	Treasures of Chinese Instrumental Music: Erhu 中国民族乐器典藏 二胡
VCEx29	Song of Melancholy 悲歌	Treasures of Chinese Instrumental Music: Erhu 中国民族乐器典藏 二胡
VCEx31	Beautiful Evening 良宵	Moon Night (10 Erhu Solo)
VCEx32	Yu Bei Ballade 豫北叙事曲	20th Century Chinese Classical Music Performances: Works of Instrumental Solo, Concert and Chorus (A)

Supplementary Results

Musical Feature Analysis

Musical features were extracted for stepwise multiple linear regression analysis using MIR Toolbox 1.8.2 [5] in MATLAB (version R2023b). Six categories with 20 features were extracted: 1) dynamics (RMS, low energy); 2) rhythm (attack time, tempo, pulse clarity, event density); 3) timbre (spectral centroid, spectral irregularity, spectral entropy, roughness, MFCC 2, spectral flux, brightness); 4) register (salient pitch); 5) tonality (key clarity and mode); and

6) musical novelty (spectral novelty, rhythm novelty, tonal novelty, register novelty). For the descriptions of the features, see Laukka, et al. [6].

A series of independent-samples *t*-tests was conducted to compare the mean differences of the musical features between the final set of Western and Chinese music stimuli. The data were transformed to *z*-scores prior to analysis. No outliers were found (< ±3.29), some Levene's tests were violated, Welch's *t* tests were reported. Some normality assumptions were violated but not addressed. Table S5 presents the feature comparison results.

Table S5

Results of Independent-samples t-Tests Comparing Musical Features between Western and Chinese Music

	t	df	*P*	M_{dif}	*SE*	95% CI	Cohen *d*
RMS M	-4.72	*t*(40.38)	<.001	-1.13	0.24	[-1.61, -0.65]	-1.36
Low energy	2.62	*t*(46)	.012	0.04	0.12	[0.01, 0.08]	0.75
Attack time M	-0.95	*t*(39.09)	.349	-0.27	0.29	[-0.86, 0.31]	-0.27
Tempo M	1.39	*t*(46)	.173	0.40	0.29	[-0.18, 0.97]	0.40
Pulse clarity	1.81	*t*(46)	.077	0.51	0.28	[-0.06, 1.08]	0.52
Spectral Centroid	1.99	*t*(46)	.053	0.56	0.28	[-0.01, 1.13]	0.57
Spectral entropy	1.83	*t*(46)	.075	0.51	0.28	[-0.05, 1.08]	0.53
Roughness	0.91	*t*(43.96)	.366	0.26	0.29	[-0.32, 0.85]	0.26
Spectral flux	-4.75	*t*(33.27)	<.001	-1.14	0.24	[-1.62, -0.65]	-1.37
Salient pitch M	-2.05	*t*(35.25)	.048	-0.57	0.28	[-1.14, -0.01]	-0.59
Key clarity	0.20	*t*(46)	.841	0.06	0.29	[-0.53, 0.65]	0.06
Mode	-0.88	*t*(46)	.384	-0.25	0.29	[-0.84, 0.33]	-0.25
Spectral novelty	0.42	*t*(46)	.674	0.12	0.29	[-0.46, 0.71]	0.12
Tonal novelty	0.27	*t*(46)	.787	0.08	0.29	[-0.51, 0.67]	0.08

Note. Raw scores converted to *z*-scores for comparison. Welsch *t* was used where applicable. Western music *n* = 24, Chinese music *n* = 24.

Musical Feature Prediction Results

A series of stepwise multiple linear regression analyses were performed to investigate whether musical features to Western and Chinese music were significant predictors of cultural classification accuracy (% correct classification) and speed (response time). Stepwise criteria included Probability-of-F-to-enter <= .050, Probability-of-F-to-remove >= .100). Stepwise methods excluded six features for lack of significant contribution. The remaining specific

features were classified into the six general categories: dynamics (RMS, low energy), rhythm (attack time, tempo, pulse clarity, event density), timbre (spectral centroid, spectral entropy, roughness, spectral flux), register (salient pitch), tonality (key clarity), and musical novelty (spectral novelty, tonal novelty). Tables S6-16 present the musical features prediction results. Western music n = 24; Chinese music n = 24; *$p < .050$; **$p < .010$; ***$p < .001$.

Table S6

Results of Stepwise Multiple Linear Regression Analysis on Musical Features Predicting Happiness Accuracy

Variables	B	β	sr^2	Adj.R^2	F	f^2
Western music				.17	5.79 *	0.26
Event density	.60 *	.46	0.21			
Chinese music				.61	12.87 ***	1.93
Tonal novelty	.36 *	.35	0.11			
Spectral novelty	.49 **	.48	0.20			
Roughness	.35 **	.42	0.17			

Table S7

Results of Stepwise Multiple Linear Regression Analysis on Musical Features Predicting Sadness Accuracy

Variables	B	β	sr^2	Adj.R^2	F	f^2
Western music				.66	12.26 ***	1.96
Pulse clarity	-.67***	-.65	0.37			
Tempo M	.53***	.49	0.22			
Key clarity	-.37*	-.34	0.11			
Spectral entropy	-.44*	-.33	0.10			
Chinese music				.78	27.87 ***	3.50

Variables	*B*	*β*	sr^2	Adj.R^2	*F*	f^2
Roughness	-.38 **	-.50	0.13			
Tonal novelty	-.38 ***	-.40	0.16			
Spectral flux	-.24 *	-.30	0.05			

Table S8

Results of Stepwise Multiple Linear Regression Analysis on Musical Features Predicting Agitation Accuracy

Variables	*B*	*β*	sr^2	Adj.R^2	*F*	f^2
Western music						
Chinese music				.94	95.32 ***	16.54
Spectral flux	1.75 ***	1.73	0.45			
RMS M	-1.25 ***	-1.14	0.19			
Spectral novelty	-0.40 ***	-.33	0.11			
Pulse clarity	.23 **	.17	0.02			

Table S9

Results of Stepwise Multiple Linear Regression Analysis on Musical Features Predicting Calmness Accuracy

Variables	*B*	*β*	sr^2	Adj.R^2	*F*	f^2
Western music				.30	6.00 **	0.57
Spectral entropy	-.42 *	-.43	0.18			
Attack time M	.45 *	.44	0.19			
Chinese music				.64	21.37 ***	1.77
Spectral flux	-2.03 ***	-1.80	0.58			
RMS	1.62 ***	1.33	0.31			

Table S10

Results of Stepwise Multiple Linear Regression Analysis on Musical Features Predicting Happiness Intensity

Variables	*B*	*β*	sr^2	Adj.R^2	*F*	f^2
Western music				.56	10.79 ***	1.36
Pulse clarity	.80 ***	.87	0.58			
Tempo M	-.39 *	-.38	0.13			
Salient pitch M	.45**	.53	0.19			
Chinese music				.55	15.26 ***	1.45
Tonal novelty	.60 ***	.64	0.44			
Low energy	-.35 **	.41	0.17			

Table S11

Results of Stepwise Multiple Linear Regression Analysis on Musical Features Predicting Sadness Intensity

Variables	*B*	*β*	sr^2	Adj.R^2	*F*	f^2
Western music				.61	12.91 ***	1.93
Pulse clarity	-.84 ***	-.73	0.49			
Tempo M	.52**	.43	0.17			
Spectral centroid	-.48 *	-.36	0.12			
Chinese music				.73	31.34 ***	2.98
Roughness	-.58 ***	-.69	0.45			
Tonal novelty	-.45 ***	-.44	0.19			

Table S12

Results of Stepwise Multiple Linear Regression Analysis on Musical Features Predicting Agitation Intensity

Variables	*B*	*β*	sr^2	Adj.R^2	*F*	f^2
Western music				.39	8.29 **	0.79
Attack time M	-.61 **	-.51	0.26			
Spectral entropy	.48 *	.43	0.19			
Chinese music				.89	38.58 ***	10.76
Spectral flux	1.46 ***	1.46	0.17			
RMS M	-1.12 ***	-1.03	0.11			
Rhythm novelty	-0.30 **	-.25	0.06			
Low energy	.27 *	.25	0.02			
Event density/s	.36 *	.37	0.03			

Table S13

Results of Stepwise Multiple Linear Regression Analysis on Musical Features Predicting Calmness Intensity

Variables	*B*	*β*	sr^2	Adj.R^2	*F*	f^2
Western music				.49	11.85 ***	1.13
Spectral entropy	-.49 **	-.51	0.26			
Attack time M	.54 **	.53	0.28			
Chinese music				.58	16.73 ***	1.59
Spectral flux	-.37 *	-.41	0.13			
Spectral entropy	-.44 **	-.51	0.20			

References

1. Eerola, T.; Vuoskoski, J.K. A comparison of the discrete and dimensional models of emotion in music. *Psychol. Music* **2011**, *39*, 18-49, https://doi.org/10.1177/0305735610362821.
2. Wang, X.; Wei, Y.; Heng, L.; McAdams, S. A cross-cultural analysis of the influence of timbre on affect perception in Western classical music and Chinese music traditions. *Front. Psychol.* **2021**, *12*, https://doi.org/10.3389/fpsyg.2021.732865.
3. Song, W.; Horner, A.B. Uncovering the differences between the violin and erhu musical instruments by statistical analysis of multiple musical pieces. *Proc. Meet. Acoust.* **2022**, *50*, 050005, https://doi.org/10.1121/2.0001710.
4. Xie, L.; Gao, Y. A database for aesthetic classification of Chinese traditional music. *Cogn. Comput. Syst.* **2022**, *4*, 197-204, https://doi.org/10.1049/ccs2.12064.
5. Lartillot, O.; Toiviainen, P.; Eerola, T. A Matlab Toolbox for Music Information Retrieval. In *Data Analysis, Machine Learning and Applications*, Preisach, C., Burkhardt, H., Schmidt-Thieme, L., Decker, R., Eds.; Springer Berlin Heidelberg: Berlin, Heidelberg, 2008; pp. 261-268.
6. Laukka, P.; Eerola, T.; Thingujam, N.S.; Yamasaki, T.; Beller, G. Universal and culture-specific factors in the recognition and performance of musical affect expressions. *Emotion* **2013**, *13*, 434-449, https://doi.org/10.1037/a0031388.
